# Divergent behavioral strategies in Wistar and Wistar-Kyoto rats in a naturalistic task reveal mood disorder phenotypes

**DOI:** 10.1016/j.isci.2026.115459

**Published:** 2026-03-21

**Authors:** Jackson R. Ham, Robert J. McDonald

**Affiliations:** 1 Department of Neuroscience, Canadian Centre for Behavioural Neuroscience, University of Lethbridge, Lethbridge, AB T1K 3M4, Canada; 2 Alzheimer Society of Canada, Toronto, ON, Canada

**Keywords:** Behavioral neuroscience, Animal science

## Abstract

Rodent models are essential for studying mood disorders, yet many assays rely on methods that may not reflect natural behavior. Food hoarding offers an ethologically relevant paradigm integrating reward seeking, risk assessment, and safety-seeking strategies. We assessed hoarding in Wistar-Kyoto (Kyoto) rats, a model of depression and anxiety, compared with Wistar controls. Kyoto rats, particularly females, displayed robust hoarding, frequently transporting all food items to the safe base while consuming little. In contrast, Wistars consumed more and showed greater exploratory activity, moving at higher velocity. Principal component analysis and k-means clustering identified distinct behavioral phenotypes, with Kyotos clustered into high hoarding, low exploratory profiles consistent with anxiety- or depressive-like profiles, while Wistars clustered into more exploratory phenotypes. Pose estimation revealed reduced locomotor velocity in Kyoto rats compared with Wistar controls, consistent with altered risk assessment. Overall, this task captures strain- and individual-level variation in mood-related behavior under more naturalistic conditions.

## Highlights

• Kyoto rats hoard more food while Wistar rats consume more during the task
• Kyoto rats show reduced exploration and slower locomotor velocity
• Multivariate analyses reveal strain-specific behavioral phenotypes
• Food hoarding captures naturalistic anxiety- and depression-like behavior

## Graphical abstract

**Figure:**
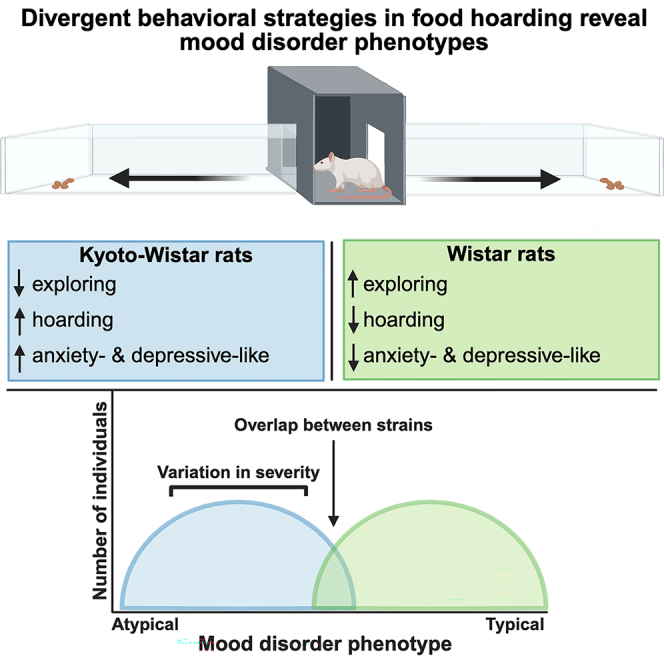

## Introduction

Etiological models of human disease are difficult to achieve in the laboratory setting, and rodent models of mood disorders are no exception.^1^^,^^2^ Despite these challenges, developing robust models of depression and anxiety, as well as appropriate assays to assess behavioral symptomology, are necessary to understand the pathology and mechanisms of disease and develop potential treatments.^3^ With depression and anxiety being some of the most debilitating disorders worldwide,^4^^,^^5^ it is critical that reliable rodent models and assays are developed. To date, some of the most reliable models of behavior take advantage of naturally occurring, ethologically relevant paradigms to test behavior.^2^^,^^6^^,^^7^

There are many rodent models of mood disorders,^8^ with some developed for anxiety-like behaviors and others for depression-like symptoms. One of the most commonly used genetic models of depression is the inbred Wistar-Kyoto rat (Kyoto).^9^ The Kyoto rat was initially bred from the Wistar rat, which is typically used as the control strain, and displays an elevated response to stress compared to other strains, including Wistar rats. For example, in more traditional tasks, Kyoto rats show immobility in the forced swim^10^ and open field tests.^11^ Kyoto rats also engage in less play—a naturally occurring behavior^12^—than Wistar rats, at all ages, demonstrating anhedonia through emotionally and socially relevant behavior.^13^ These studies, and others (e.g.,^14^^,^^15^^,^^16^^,^^17^^,^^18^^,^^19^) suggest that Kyoto rats show both anxiety-like and depressive-like behaviors when tested with traditional and non-traditional assays. Traditional assays such as forced swim, open field, and sucrose preference tests have been invaluable for characterizing anxiety- and depression-like behavior in rodents. However, comparatively less work has examined how these behavioral phenotypes manifest in more naturalistic contexts, such as food hoarding.

Food hoarding provides a particularly valuable framework for examining mood-related behaviors in rodents because it integrates multiple motivational and affective domains.^20^^,^^21^^,^^22^^,^^23^ In the food hoarding task, to obtain food items, rats must leave a secure, enclosed space and traverse an open area, a decision that reflects risk assessment and anxiety-like behavior.^24^^,^^25^ Once food is obtained, rats may either consume it immediately or transport it back to safety, a choice that may reflect differences in reward-directed behavior and strategies for navigating potentially threatening environments^22^^,^^23^^,^^26^^,^^27^ and is also observed in wild rats.^28^ Alternatively, it could reflect the internal mood state of the animal (e.g.,^29^^,^^30^^,^^31^). Finally, the refusal to exit the safe base or engage with available food can be interpreted as a depressive-like avoidance phenotype, whereas hoarding behavior itself is considered an anxiety-related safety-seeking strategy. Unlike traditional paradigms that typically isolate a single dimension—such as behavioral despair in the forced swim test^10^ or consummatory anhedonia in sucrose preference^32^—food hoarding encompasses several processes simultaneously, offering a richer, ecologically grounded picture of behavioral strategies in different strains. One limitation with food hoarding, however, is that hoarding is not inherently pathological and may also reflect adaptive risk-management strategies, differences in motivation, or valuation of the food reward. Additionally, although rats may initially exhibit hesitancy when engaging in food hoarding, the laboratory environment does not impose energetic scarcity or survival pressure and therefore does not require animals to balance immediate consumption against long-term food security. As a result, the absence of hoarding may be more indicative of maladaptive avoidance or depressive-like disengagement in this context.

Despite extensive use of the Kyoto rat in traditional behavioral assays (e.g.,^10^^,^^15^^,^^16^^,^^18^^,^^19^^,^^33^), little is known about how this strain engages in naturalistic, ethologically relevant behaviors such as hoarding. Classical tests have provided a strong foundation for characterising depressive- and anxiety-like phenotypes,^9^^,^^34^ but comparatively fewer studies have extended these observations to complex behaviors that may better approximate how mood disorders influence daily activities.^13^ By investigating food hoarding, we aim to bridge this gap and determine whether the well-documented anxiety- and depression-like traits of Kyoto rats extend to a naturalistic context that requires balancing risk, reward, and safety.

The present study, therefore, examined food hoarding behavior in Kyoto rats compared with Wistar controls. Based on the well-documented anxiety- and depression-like traits of the Wistar-Kyoto strain,^9^^,^^34^ we predicted that these rats would differ from controls in several aspects of the task. We predicted they would take longer to leave the safe base, reflecting heightened avoidance, consume fewer food items in the open area, which could reflect anxiety-related avoidance, altered motivation, or reduced reward salience, and transport more food back to the safe base, reflecting anxiety-driven strategies. Alternatively, some Kyoto rats may fail to engage in the task altogether, a pattern more consistent with severe depressive-like behavior. By testing these possibilities, our study aims to clarify how mood-related traits manifest in a naturalistic paradigm and to evaluate the utility of food hoarding as a complementary, multidimensional assay that captures behavioral variation not easily assessed by traditional paradigms, at both the strain- and individual-level, thereby providing a foundation for future mechanistic investigations.

## Results

### Behavioral differences in food hoarding among strains

**Figure 1:**
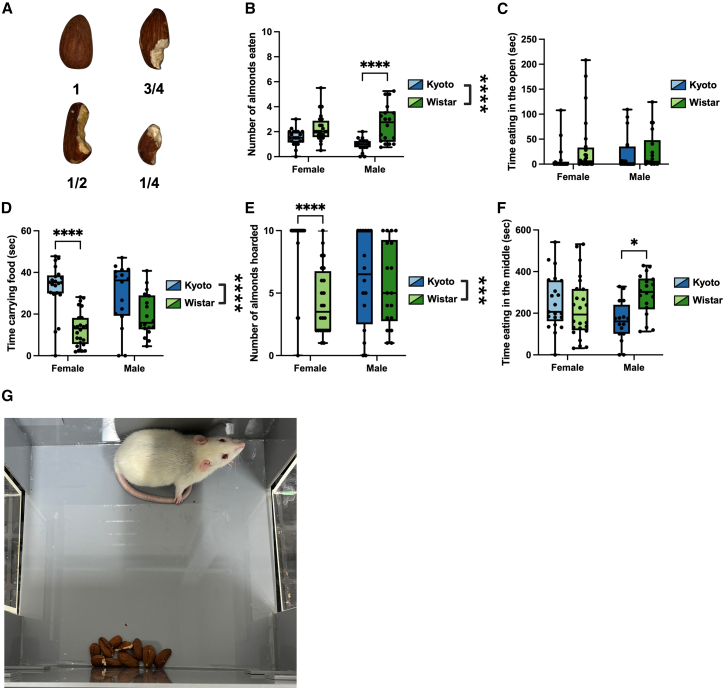
Strain- and sex-dependent differences in food hoarding and consumption Kyoto rats, particularly females, preferentially hoarded almonds rather than consuming them, whereas Wistar rats ate more and explored the apparatus more actively. (A) Almonds were scored based on the proportion consumed (whole, ¾, ½, and ¼). (B) Wistar rats consumed significantly more almonds than Kyoto rats, with a pronounced effect in males. (C) No strain or sex differences were observed in time spent eating in the open arms. (D) Kyoto rats, especially females, spent more time carrying almonds than Wistar rats. (E) Kyoto females hoarded significantly more almonds than Wistar females, with most Kyoto females hoarding all 10 almonds provided. It should be noted that a rat could have eaten some parts of the almond without hoarding any of the almonds. (F) Male Wistar rats spent more time eating in the middle chamber than Kyoto males. (G) Representative image of a Kyoto female rat arranging hoarded almonds into piles in the middle chamber. Data are shown as boxplots with individual points ± max and min and tested with two-way ANOVAs; ∗*p* < 0.05 and ∗∗∗∗*p* < 0.0001.

Following trials, the number of almonds eaten was counted, noting how much of each almond was consumed (Figure 1A). Wistar rats ate more almonds than Kyoto rats overall (Figure 1B), with male Wistar rats consuming more than Kyoto males (Table S1). No differences were observed in the time spent eating almonds in the open (Figure 1C), but male Wistar rats spent more time eating in the middle chamber compared to Kyoto males (Figure 1F) (Table S1). Kyoto rats spent more time carrying almonds than Wistar rats (Figure 1D), with Kyoto females spending more time carrying almonds than Wistar females (Table S1). We also observed a strain difference in the number of almonds hoarded (Figure 1E), defined as the number of almonds taken back to the middle chamber (Table S1). Kyoto females hoarded significantly more almonds than Wistar females, with nearly all Kyoto females (18/21) hoarding all 10 almonds provided (Figure 1E). Interestingly, despite this tendency to hoard, the females did not consume as many almonds; after collecting them, the rats often arranged the almonds into neat piles in the middle chamber (Figure 1G). Overall, these results indicate that Kyoto rats, particularly females, preferentially hoard rather than consume food, whereas Wistar rats are more likely to eat the almonds directly.

**Figure 2:**
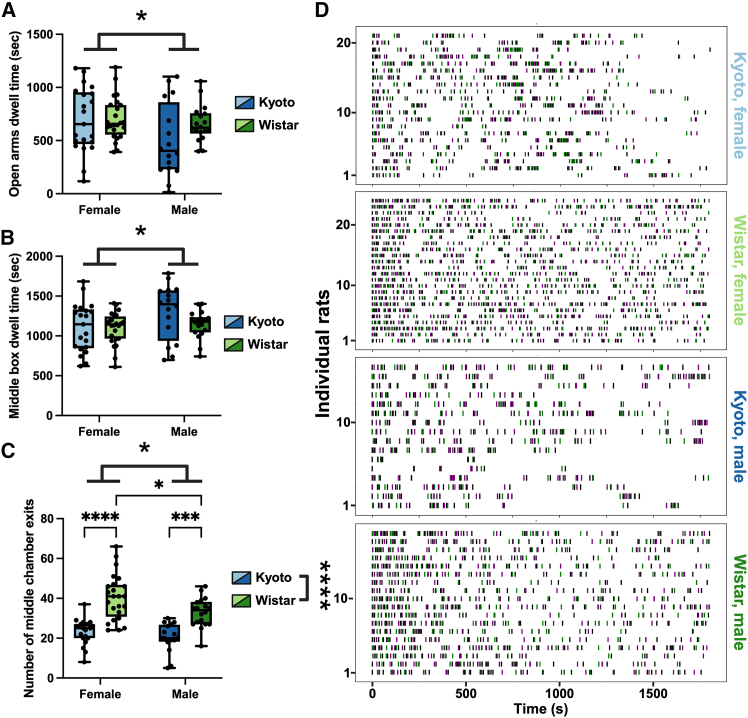
Strain- and sex-dependent differences in exploratory behavior (A) Females, regardless of strain, spent more time in the arms of the apparatus. (B) Males, regardless of strain, spent more time in the middle chamber. (C) Number of exits from the middle chamber revealed significant sex, strain, and sex × strain effects: Wistar females exited more than Kyoto females and Wistar males, and Wistar males exited more than Kyoto males. (D) Raster plots show that Wistar rats, regardless of sex, moved more frequently between chambers compared to Kyoto rats. Left arm exits are plotted in green, middle exits in black, and exits from the right arm exits in purple. Together, these data indicate that Kyoto rats displayed reduced exploratory activity, particularly females, whereas Wistar rats were more active and engaged in more frequent chamber transitions. Data are shown as boxplots with individual points ± max and min and tested with two-way ANOVAs; ∗*p* < 0.05, ∗∗∗*p* < 0.001, and ∗∗∗∗*p* < 0.0001.

While females, regardless of strain, spent more time in the arms (Figure 2A) and males spent more time in the middle box (Figure 2B), we found a sex, strain, and sex × strain difference (Figure 2C) in the number of times the rats exited the middle chamber (Table S1). Wistar females showed the highest number of middle-chamber exits, exceeding both Kyoto females and Wistar males (Figure 2C). Raster plots reveal that Wistars, regardless of sex, were moving through the apparatus more than Kyoto rats (Figure 2D).

Additionally, while time spent exploring (i.e., time in the open arms) was positively correlated with the number of almonds hoarded in both male and female Kyoto rats and male Wistar rats, we did not find a significant correlation between exploration time and the number of almonds hoarded for female Wistar rats (Table S1). Together, these results indicate strain- and sex-dependent differences in hoarding, consumption, and exploratory engagement during the task.

### Strain differences in anxious- and depressive-like behavioral phenotypes

#### Behavioral phenotypes identified by clustering

**Figure 3:**
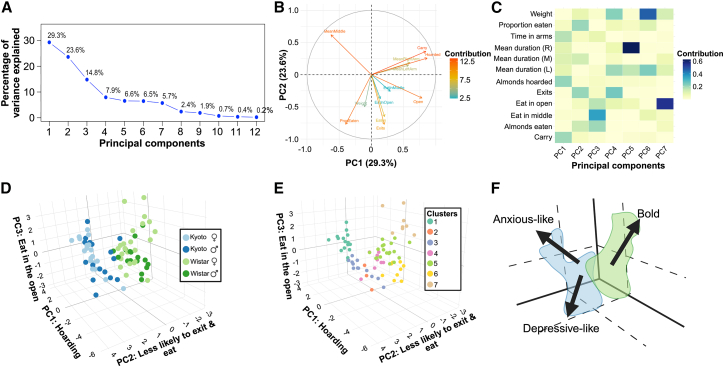
Principal component analysis and behavioral clustering of rat phenotypes (A) Scree plot shows the percentage of variance explained by each of the 12 principal components (PCs). The first seven PCs capture the majority of the variance in the dataset. (B) PCA correlation circle plot illustrates the contributions of individual behavioral variables to PC1 and PC2. Arrows indicate the direction and strength of each variable’s contribution, with color representing the magnitude of the contribution. (C) Heatmap of squared loadings (contributions) of 12 behavioral variables to the first seven PCs. Darker colors indicate higher contributions, highlighting which behaviors drive variance along each component. (D) 3D PCA plot of PC1–PC3 shows the separation of Kyoto and Wistar rats in behavioral space. Kyoto rats cluster together (blue), while Wistar rats form a distinct cluster (green). (E) K-means clustering on the first three PCs identifies seven behavioral phenotypes. Each color represents a different cluster, with cluster composition summarized in Table S2. (F) Summary of strain- and sex-dependent behavioral profiles for each cluster. Kyoto rats generally exhibit high hoarding (anxious-like) and reduced exploration (depression-like), whereas Wistar rats show more exploratory and bold phenotypes with lower hoarding.

Principal Component Analysis (PCA) of the 12 behavioral measures revealed that the first three principal components captured the majority of variance in the dataset (PC1: 29.3%, PC2: 23.6%, PC3: 14.8%; scree plot shown in Figure 3A). Examination of variable loadings indicated that PC1 was primarily driven by hoarding-related behaviors (i.e., carrying almonds and number of almonds hoarded) and PC2 reflected exploratory behavior and propensity to exit the safe zone (Figure 3B).

A heatmap of squared loadings (Figure 3C) further highlighted variable contributions to the first seven PCs. Hoarding-related measures (i.e., carry and number of almonds hoarded) contributed most strongly to PC1, consistent with its association with anxiety- or depressive-like behaviors. Exploratory measures (i.e., exits and open) and eating in open areas loaded prominently on PC2 and PC3, capturing variance related to bold or exploratory phenotypes. Subsequent PCs (PC4–PC7) captured additional, subtler behavioral variation across the dataset. Together, the heatmap and correlation circle provide a clear view of how individual behaviors influence the multivariate structure.

When the first three PCs were plotted in 3D space (Figure 3D), Kyoto and Wistar rats occupied partially separable regions of PCA space, with some overlap between strains. Kyoto rats clustered together, exhibiting increased hoarding, reduced eating in the open, and fewer exits from the middle chamber, while Wistar rats occupied a separate region, reflecting higher exploratory activity and food consumption in open zones. Within each strain, males and females overlapped substantially, indicating that strain exerted a stronger influence on overall behavioral profiles than sex.

The number of clusters (*k* = 7) was selected using silhouette analysis, which identified this solution as maximizing cluster separation while minimizing overlap. Importantly, the major behavioral phenotypes were preserved across adjacent *k* values, indicating that the clustering solution was robust to reasonable variation in cluster number. K-means clustering on the first three PCs identified seven behavioral clusters (Figure 3E; Table S2). Clusters 1–3 were dominated by Kyoto rats. Cluster 1 consisted primarily of Kyoto females exhibiting high hoarding, extensive carrying, and low exploratory behavior (**anxiety-like** **phenotype**). Cluster 2 contained a mix of Kyoto males and females with minimal eating, low carrying, and very few exits (**depressive-like phenotype**). Cluster 3 included equal numbers of Kyoto males and females with moderate exploratory activity, occasional eating, and hoarding (more Wistar **typical** **behavior**).

Clusters 4–7 were largely composed of Wistar rats with varying sex distributions. Cluster 4 included both Wistar males and females with high exploratory behavior, frequent exits, and moderate eating and hoarding. Cluster 5 consisted mainly of Wistar females showing substantial eating in middle and open areas, moderate carrying, and moderate hoarding. Cluster 6 contained mostly Wistar males with frequent exits, substantial eating in the middle chamber, moderate carrying, and low hoarding. Cluster 7 was dominated by Wistar females exhibiting high exploratory behavior, frequent arm movement, moderate eating in the open, and low hoarding (**bold phenotype)**.

Overall, these clusters suggest that behavioral phenotypes were partially strain-dependent (Figure 3F). Kyoto rats grouped into clusters characterized by high anxiety- or depressive-like behaviors (reduced exploration, high hoarding vs. reduced exploration, low hoarding, low interest in food), whereas Wistar rats formed clusters with more exploratory or bold phenotypes and lower hoarding behavior. The combination of PCA heatmap, correlation circle, and 3D PCA visualization provides mechanistic insight into which behaviors drive these strain- and cluster-dependent phenotypes.

### Velocity and postural analysis

**Figure 4:**
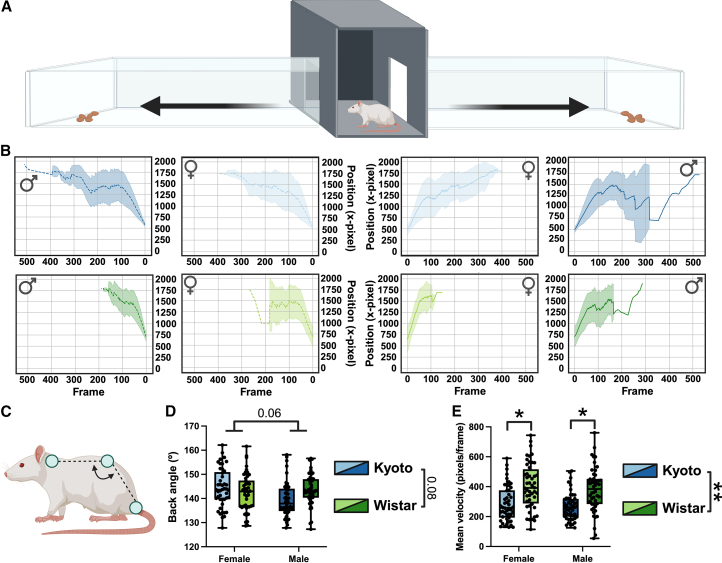
Strain differences in velocity and posture during exploratory trials (A) Schematic of the apparatus illustrating almond placement and arm traversal. (B) Trajectories from DeepLabCut tracking of Wistar (green) and Kyoto (blue) rats, separated by sex. The mean trajectory over time, for all animals, is plotted with the shaded areas representing ±1 standard deviation. (C) Wistar rats exhibited significantly higher mean velocity compared to Kyoto rats, regardless of sex. (D) Body landmarks used for posture analysis. (E) Average back angle revealed a trending effect of strain and sex, with Wistar rats tending toward greater extension. Together, these results indicate that Wistar rats moved through the apparatus more quickly and showed subtle postural differences compared to Kyoto rats. Data are shown as boxplots with individual points ± max and min and tested with linear mixed models; ∗*p* < 0.05 and ∗∗*p* < 0.01.

Using DeepLabCut, we analyzed how rats traversed the left and right arms (Figure 4A) during their first three explorations from the middle chamber to the end of the arm. Wistar rats reached the end of the arm more quickly than Kyoto rats, regardless of sex or arm (Figure 4B). This observation was supported by a significant main effect of strain on mean velocity, with pairwise comparisons showing that both male and female Wistar rats exhibited higher mean velocities than their Kyoto counterparts (Figure 4C). We also compared the average back angle of the rats while navigating the arms (Figure 4D) and observed a trending effect of both sex and strain × sex, with Kyoto females showing the flattest back angle (Figure 4E). Wistar rats not only moved through the apparatus more rapidly than Kyoto rats. Although back-angle differences did not reach statistical significance, our analyses suggest potential strain- and sex-dependent variation in posture that warrants further investigation, as the slowed movement and increased stretch-attend and stretch-approach posture suggest that the Kyoto females, in particular, engage in more risk assessment behaviors, a behavioral correlate of anxiety.^35^^,^^36^^,^^37^^,^^38^

## Discussion

In this study, we compared the food hoarding and exploratory behavior of Wistar and Kyoto rats using a food hoarding task. We found that Kyoto rats, particularly females, exhibited strong hoarding tendencies, often collecting all available almonds but consuming relatively few of them. In contrast, Wistar rats were more likely to eat almonds directly and showed higher overall exploratory activity, exiting the middle chamber more frequently and moving through the apparatus with greater velocity. Multivariate analyses reinforced these differences, with principal component and clustering approaches identifying strain-dependent behavioral phenotypes: Kyoto rats grouped into clusters characterized by high hoarding or low exploration (profiles reflecting altered risk-reward trade-offs and avoidance, consistent with anxiety- or depressive-like behavior), whereas Wistar rats clustered into phenotypes marked by greater exploration, direct food consumption, and boldness.

These findings underscore the utility of this food hoarding task as a sensitive tool for detecting strain and individual differences in anxiety- and depression-related phenotypes. Importantly, the clustering analyses demonstrated that not all animals within a strain conformed to the same behavioral profile. Some Kyoto rats displayed extreme hoarding and withdrawal, while others showed more moderate or Wistar-like patterns of behavior. Similarly, Wistars expressed both exploratory-bold and more cautious phenotypes. This heterogeneity parallels human psychiatric conditions,^39^^,^^40^ where diagnostic categories often encompass diverse symptom profiles, with comorbid symptoms,^41^^,^^42^^,^^43^ suggesting that this task may serve as a useful platform for identifying individual variability and resilience.

An additional feature revealed by the raster plots was the temporal structure of exploration. Kyoto rats often exited the safe chamber early in the session but showed a rapid decline in exploration as the session progressed. While one might expect peak avoidance at initial exposure to a novel environment, this pattern may instead reflect rapid disengagement or altered cost-benefit evaluation following early sampling of the task. Such early exploration followed by withdrawal has been observed in models of anxiety and depression^44^ and may reflect behavioral inflexibility or negative updating of perceived reward value rather than reduced initial fear. Alternatively, Kyoto rats may determine that repeated excursions are not worth the associated risk, consistent with altered motivation or reward valuation, both being behavioral phenotypes of mood disorders. These interpretations are not mutually exclusive and highlight the importance of considering temporal dynamics when interpreting naturalistic behavior.

Unlike prior protocols,^24^^,^^25^ we did not food-restrict or habituate the rats to the apparatus. Food restriction can artificially amplify motivation to seek out and hoard food,^45^^,^^46^^,^^47^^,^^48^ potentially obscuring more subtle differences in reward- or anxiety-related behavior. Likewise, habituation reduces novelty and risk,^49^^,^^50^^,^^51^^,^^52^ limiting opportunities to observe avoidance and safety-seeking. By maintaining standard feeding conditions and introducing rats to a novel environment, we think we captured a more ethologically relevant expression of hoarding.

An important alternative interpretation of the present findings concerns motivation and food valuation. Because the rats were neither food-restricted nor tested for home-cage consumption of almonds, reduced exploration or consumption in Kyoto rats could reflect lower incentive salience of the reward rather than anxiety or depressive-like states. However, lowered salience of the reward or anhedonia is commonly reported in depression.^17^ More broadly, differences in task engagement may also reflect variation in curiosity, novelty seeking, or other intrinsic motivational states that are not specific to anxiety or depression, though reduced curiosity and novelty can also be associated with mood disorders.^53^^,^^54^ Future studies incorporating home-cage feeding tests or pharmacological manipulations will be necessary to disentangle these possibilities. Importantly, the present design was intended to capture spontaneous behavioral strategies under low motivational constraint, rather than isolate hunger-driven motivation per se. However, when animals are not hungry, reluctance to enter a potentially risky environment may represent an adaptive cost-benefit decision, rather than maladaptive avoidance. In addition, testing was conducted during the light phase under standard laboratory illumination. While this is consistent with many behavioral studies, and Long Evans rats do not hoard differently depending on the illumination of the enclosure,^55^ future work conducted during the dark phase under dim red light would help clarify the contribution of circadian or sensory factors to task performance and motivation.

The ethological basis of hoarding in rats also provides an important interpretive context. Unlike hamsters, which hoard to stockpile food,^47^^,^^56^^,^^57^ rats typically carry food items to safe areas as a risk-avoidance strategy.^28^^,^^47^^,^^58^^,^^59^ Hoarding is therefore more likely to occur when the environment feels insecure,^60^^,^^61^ and anxiolytic treatment has been shown to reduce hoarding while increasing immediate consumption.^20^^,^^21^ In contrast, depressive-like phenotypes are less likely to engage with the task altogether, including minimal exploration, refusal to exit the covered base, and avoiding interaction with the available food. Our observation that Kyoto females hoarded nearly all available food items while consuming little is consistent with this framework, suggesting heightened avoidance and safety-seeking. In contrast, Wistar rats’ tendency to consume almonds directly aligns with greater boldness and reduced anxiety-like behavior.

The inclusion of both sexes revealed further meaningful differences. Female Kyoto rats were especially prone to hoarding, while female Wistars were more exploratory and consumed more food in open zones. These patterns mirror broader evidence for sex differences in affective disorders,^62^^,^^63^^,^^64^ where females have a higher prevalence of anxiety.^65^ Although the effects were modest and did not reach significance thresholds, postural analyses suggested a trend for females to more frequently adopt stretch-attend and stretch-approach postures. These postures are commonly interpreted as indices of risk assessment, which is often associated with anxious behavior.^35^^,^^36^^,^^37^^,^^38^ In the present study, they should be viewed as exploratory signals rather than definitive markers of anxiety-like behavior. Limited statistical power and technical constraints likely reduced sensitivity to detect robust group differences. Nonetheless, these findings underscore the importance of considering sex as a biological variable^66^ and suggest that Kyoto females, in particular, may be a valuable model for maladaptive avoidance behaviors.

That Kyoto females hoarded extensively without consuming the food raises the possibility that hoarding and eating are governed by partially dissociable processes. While hoarding may reflect a rapid, risk-averse strategy to secure resources, immediate consumption in the open arena requires prolonged exposure and sustained reward valuation. Thus, this pattern could also reflect behavioral inflexibility, sensory-specific satiety, or altered reward processing, all of which are symptoms of an anxiety or depressive-like state. Disambiguating these possibilities will require future experiments assessing food valuation and consumption in low-risk contexts.

The stronger phenotype observed in females may also reflect hormonal or neuroendocrine influences. Sex differences in hypothalamic-pituitary-adrenal (HPA) axis regulation, stress reactivity, and gonadal hormone signaling have been implicated in increased anxiety- and depression-related vulnerability in females.^67^^,^^68^^,^^69^ Fluctuations in ovarian hormones can modulate risk assessment, avoidance, and reward valuation, and may therefore contribute to the pronounced hoarding and cautious exploratory strategies observed in Kyoto females.^70^^,^^71^

An important future direction for this paradigm is pharmacological validation.^20^ The Wistar-Kyoto strain has been widely characterized as a model of treatment-resistant depression,^72^^,^^73^ showing limited responsiveness to conventional antidepressants such as imipramine but sensitivity to rapid-acting agents such as ketamine.^74^^,^^75^ Applying such pharmacological manipulations within the food hoarding task could help determine whether naturalistic measures of avoidance, hoarding, and exploration are sensitive to clinically relevant treatment effects. This would further enhance the translational utility of the paradigm by linking ethologically expressed behavioral strategies to pharmacological responsiveness.

### Limitations of the study

Despite its strengths, the food hoarding task has caveats that should be considered when interpreting the present findings. First, hoarding behavior is inherently multidimensional and may reflect overlapping motivational processes, including anxiety-related avoidance, depression-related altered reward valuation, risk sensitivity, or energy conservation. A related limitation of the present design is that differences in motivation or food valuation cannot be fully dissociated from anxiety-related avoidance. Addressing this distinction would require additional assays (e.g., home-cage food consumption or preference measures), which were beyond the scope of the current study. Additionally, because no food-restricted comparison group was included, the present design cannot directly address how food restriction modulates hoarding, exploration, or strain differences in this task. Second, performance in the task is sensitive to contextual variables such as environmental novelty, lighting conditions, and apparatus design, which complicate direct comparisons across laboratories. Third, although the absence of food restriction may enhance ethological validity, it may reduce task engagement or sensitivity in some animals with low baseline motivation (though variation in baseline motivation might reflect underlying mood disorders), potentially underestimating group differences in consumption-related measures. Fourth, a further limitation is that by focusing on early traversals, the present postural and movement analyses do not address how behavioral strategies evolve over time or whether strains differ in habituation to the task. Future work examining full-session dynamics or longitudinal changes in exploration and hoarding will be necessary to determine whether Wistar and Kyoto rats differ in their rate or pattern of habituation. Finally, strain- and sex-dependent behavioral strategies, while informative, introduce additional complexity when interpreting group-level effects and underscore the value of multivariate approaches. Together, these considerations emphasize that food hoarding should be viewed as a complementary, rather than substitutive, assay that captures naturalistic behavioral strategies not easily assessed using traditional paradigms.

A further limitation concerns the sensitivity of the automated postural analyses, particularly for stretch-attend and stretch-approach behaviors. These postures are subtle and transient, which may have reduced tracking accuracy. In addition, only a limited set of body parts was tracked, constraining the precision with which back angle and body elongation could be estimated. Future work could increase statistical power and measurement fidelity by incorporating additional body landmarks (e.g., shoulders, hips, and tail base) and using multiple camera placements. Such refinements would improve the detection of postural differences and allow more definitive conclusions regarding risk assessment behaviors.

Taken together, these results establish the food hoarding task as a powerful diagnostic tool for assessing variation in anxiety- and depression-like phenotypes in rodents. By combining direct measures of hoarding, exploratory activity, and automated posture and velocity tracking, the task provides a multidimensional view of behavior without relying on artificial motivational manipulations. This approach holds promise for probing the mechanisms of vulnerability and resilience,^76^ testing therapeutic interventions,^20^^,^^21^ and advancing the translational relevance of rodent models of affective disorders.^77^ We believe this paradigm would complement other standard behavioral assays, providing an ethologically grounded perspective on motivational and affective strategies. Future work incorporating pharmacological validation, as well as correlating the behavior observed in the food hoarding task with more traditional tasks, such as the elevated plus maze, could further establish the translational relevance of this paradigm.

## Resource availability

### Lead contact

Further information and request for resources should be directed to the lead contact, Jackson R. Ham (jackson.r.ham@gmail.com).

### Materials availability

This study did not generate new unique reagents.

### Data and code availability

• All the raw data used for statistical analyses have been deposited on figshare and are publicly available (https://doi.org/10.6084/m9.figshare.30131980).
• The code used for statistical analyses has been deposited on figshare and is publicly available (https://doi.org/10.6084/m9.figshare.30131980).
• Any additional information required to reanalyze the data reported in this paper is available from the lead contact upon request.

## Acknowledgments

Thank you to Vannessa Urlacher for breeding the animals and to the animal care staff at the Canadian Centre for Behavioural Neuroscience for diligent care of the animals. We also thank Dr. Sergio Pellis for his advice on the food hoarding paradigm. This study was supported by the 10.13039/100008740University of Lethbridge Research Fund (RJM and JRH), the Natural Sciences and Engineering Council of Canada (RJM: RGPIN-2020-06929; JRH: CGS-D), the Canadian Institutes of Health Research (RJM: PJT-175091), and the 10.13039/501100000143Alzheimer Society of Canada (JRH: 10.13039/100012498ASRP).

## Author contributions

J.R.H.: conceptualization, formal analysis, methodology, visualization, writing – original draft, writing – review and editing, and funding acquisition. R.J.M.: conceptualization, formal analysis, methodology, supervision, visualization, writing – original draft, writing – review and editing, and funding acquisition.

## Declaration of interests

The authors declare no competing interests.

## STAR★Methods

### Key resources table

**Table:** 

REAGENT or RESOURCE	SOURCE	IDENTIFIER
**Depositeddata**		

Raw data and code	This paper	Figshare (https://doi.org/10.6084/m9.figshare.30131980)

**Experimentalmodels:Organisms/strains**		

Rat/Wistar (RRID: RGD 13508588)	Charles River Laboratories	N/A
Rat/Wistar Kyoto (RRID: RGD 1358112)	Charles River Laboratories	N/A

**Softwareandalgorithms**		

R Studio (version 4.4.3)	https://cran.r-project.org	https://cran.r-project.org
Python (version 3.11)	https://www.python.org/	https://www.python.org/
DeepLabCut (version 3.0.0rc8)	https://www.mackenziemathislab.org/deeplabcut	https://www.mackenziemathislab.org/deeplabcut
GraphPad Prism (version 10.2.3)	https://www.graphpad.com/	https://www.graphpad.com/
BORIS	https://www.boris.unito.it/	https://www.boris.unito.it/

### Experimental model and study participant details

#### Subjects

Outbred Wistar (24 females and 18 males) and inbred Wistar-Kyoto (Kyoto) (21 females and 16 males) rats were bred at the Canadian Centre for Behavioural Neuroscience. When the pups were 21 days of age, they were weaned and placed into separate, same-strain, same-sex pairs. All rats had food (irradiated PicoLab 5LOD, LabDiet) and water available *ad libitum*. The rats were housed on a 12-h light-dark cycle (lights on between 0730 and 1930) in a room maintained at a constant temperature of 21°C–23°C.

All care and testing procedures were approved by the University of Lethbridge Animal Welfare Committee (Protocols: 2307 and 2306, breeding and experimental protocols, respectively) in compliance with guidelines from the Canadian Council for Animal Care.

### Method details

#### Apparatus

Food hoarding was conducted in a two-arm maze.^24^ Both arms (91 × 15 × 15 cm) were constructed of clear Plexiglas® with a removable clear lid and protruded from a center “safe base.” The center base (30 × 30 × 30 cm) was constructed of opaque Plexiglas® that was gray in color. The center base had openings on either side (15 × 15 cm) that allowed the rat to enter the arms. The center base was sealed with an opaque gray lid. For an illustration, see Figure 4A.

Trials were recorded with a Sony Handycam FDR-AX53 digital camera which was placed in-line with the testing apparatus. The camera’s vertical position was adjusted to be level with the apparatus to ensure that the entire setup was captured on video.

#### Procedure

When the rats were around 140 days of age, the food hoarding trials began. Unlike previous reports,^24^^,^^25^ we did not food restrict our animals or habituate the animals to the enclosure or the food. This design choice ensured that behaviour in the task reflected baseline motivational and affective states, rather than being influenced by hunger or prior familiarity with the apparatus.

Rats were moved from their home cages into transfer cages and taken to the test room where they were placed into the center base. Before placing the rats into the apparatus, five unsalted almonds were placed at the end of each arm. Almonds were selected as a palatable, manipulable food item that reliably elicits carrying behavior in rodents,^78^ although strain differences in food valuation cannot be excluded. The rats were naïve to almonds. The rats were left to freely explore the apparatus for 30 min. Following the trial, the rats were removed from the enclosure, the almonds were removed and discarded, and the apparatus was cleaned with Virkon® and reset for the next animal. A limitation of this design is that differences in motivation or food valuation cannot be fully dissociated from anxiety-related avoidance. Addressing this distinction would require additional assays (e.g., home-cage consumption), which were beyond the scope of the present study.

### Quantification and statistical analysis

#### Behavioral analysis

Time spent in the left arm, right arm, and middle chamber was scored from the moment both forepaws and shoulders crossed the doorway threshold into one of the chambers until the moment both forepaws and shoulders crossed back out of that chamber. In addition, to determine how frequently the rats were leaving the middle chamber to explore, the number of middle chamber exits were calculated. The mean time spent in each arm and the middle chamber was also calculated. Following previous reports,^24^^,^^25^ we calculated the time the rats spent carrying the almonds. Time spent carrying the almonds was accumulated from the moment the almond was placed in the mouth until the almond fell or was removed from the mouth. Carrying time was incurred when devoid of eating. Time spent eating in either the arms or in the middle was also measured. Durations were measured using the open-source program BORIS.^79^

In addition to durations, following each observation, the number of almonds eaten and the number of almonds hoarded was noted. Almonds were classified as hoarded if they were carried all the way back to the center chamber.

#### DeepLabCut

##### Video acquisition

Behavioral sessions were recorded using a Sony Handycam FDR-AX53 4K camera at 30 frames per second under uniform white lighting. Each trial included multiple arm explorations, so, for analysis, we trimmed the original recordings of the session to only include the first three complete traversals from the middle chamber to the ends of the left and right arms for each animal, resulting in six videos per animal. This window was selected to capture initial exploratory behavior, when novelty and perceived risk are highest. Restricting analyses to a fixed number of early traversals also standardized the analyzed period across animals and reduced variability associated with differences in total session duration or late-session disengagement. In addition, limiting the analysis window reduced computational demands associated with pose-estimation and kinematic analyses. In addition, videos were trimmed so that animals were fully within the open arms, where body landmarks were not occluded by the center chamber. Eight animals per sex and strain (Wistar and Wistar-Kyoto) were included. Before processing with DeepLabCut, videos were cropped to just show the pertinent arm and traversal and stored in MP4 format.

##### DeepLabCut setup and training

Markerless pose estimation was performed using DeepLabCut^80^ (version 3.0.0rc8). From 8 videos (1 per sex/strain/arm), 20 frames (a total of 160 frames) were manually labeled using k-means frame selection to maximize coverage of the animals’ postures. The labeled body parts included the nose, nape of the neck, middle of the back, and base of the tail. An initial set of eight videos was used to train the network. Labeled data were split into training and test sets using the default DeepLabCut training fraction (95% training, 5% test). Model performance was evaluated using DeepLabCut’s evaluation tools. Training RMSE was 4.6 pixels (3.91 pixels after likelihood thresholding), and test RMSE was 7.43 pixels (6.62 pixels after thresholding). Mean average precision and recall exceeded 99% for both training and test datasets. The network used a ResNet50 backbone and was trained for 200 epochs with a learning rate of 1e-5. Training loss converged to 0.00176, and validation loss reached 0.00424, indicating accurate pose estimation of the labeled body parts.

##### Post-processing of DLC data

To ensure data quality, frames with a likelihood below 0.7 were excluded for back angle calculations, and a threshold of 0.9 was applied for velocity calculations. Missing or low-likelihood points were interpolated and smoothed using forward- and backward-filling to generate continuous trajectories.

##### Behavioral analysis

Nose X-positions were extracted to calculate instantaneous velocity, while the positions of the nape, back, and tail were used to calculate back curvature angles. The processed data were grouped by strain, sex, and left vs. right arm to generate summary statistics, including mean and standard deviation per frame. Custom Python scripts were used to automate the extraction, cleaning, and calculation steps, ensuring consistent data processing across all animals.

#### Statistical analysis

##### Behavioral measures

To assess strain- and sex-dependent differences in food hoarding and related behaviors, we analyzed multiple measures of task performance, including: number of almonds eaten, time spent eating in the open, time spent carrying food, number of almonds hoarded, time spent eating in the middle, time spent in the open arms, time spent in the middle chamber, and the number of exits from the middle chamber. Each behavioral measure was analyzed separately using two-way ANOVAs with strain (Wistar, Kyoto) and sex (male, female) as independent factors. All behavioral analyses were performed in GraphPad Prism (version 10.2.3), and interaction effects between strain and sex were examined. Where significant main or interaction effects were detected, post hoc pairwise comparisons were conducted using Tukey’s test. Data are plotted as mean ± min-max with asterisks used to indicate significance (∗p < 0.05, ∗∗ p < 0.01, ∗∗∗p < 0.001, ∗∗∗∗p < 0.0001).

To visualize the temporal pattern of animals’ movements within the apparatus, raster plots were generated for each rat, showing every instance they entered or exited the left arm, right arm, or middle chamber. Behavioral event data were extracted from video scoring spreadsheets and analyzed in Python (version 3.11) using pandas, Matplotlib, and matplotlib.patches. Only “START” events were included, representing the onset of each behavior. Each animal was assigned a horizontal row, and vertical lines were plotted at the corresponding time of each behavioral event, with distinct colors representing the different zones (left arm: green, middle chamber: black, right arm: purple). Separate raster plots were created for each strain × sex combination, providing a visual summary of the timing and frequency of movements across animals and groups.

##### Principal component analysis (PCA)

To reduce the dimensionality of the behavioral dataset and identify major axes of variation, PCA was performed on 12 behavioral variables including exits from the safe zone, food hoarding, consumption in different zones, and arm-specific movement metrics. PCA was conducted using the prcomp function in R^81^ (version 4.4.3) with centering and scaling enabled. The proportion of variance explained by each principal component (PC) was calculated, and a scree plot was generated to visualize the contribution of each component. Principal component retention was guided by a broken stick model, with components exceeding broken stick expectations considered non-random. The first three principal components satisfied this criterion and accounted for the majority of explained variance; these components were therefore retained for subsequent analyses. Principal component retention was guided by a broken stick model, with components exceeding broken stick expectations considered non-random. The first three principal components satisfied this criterion and accounted for the majority of explained variance; these components were therefore retained for subsequent analyses. Variable loadings were examined to interpret the behavioral dimensions represented by each PC. To provide a quantitative summary of variable contributions across multiple PCs, a heatmap of squared loadings (contributions) was created for the first seven PCs, highlighting which behaviors contributed most strongly to each component. The first three PCs, which captured the majority of variance in the dataset, were used for downstream analyses.

###### Cluster analysis/phenotype identification

To explore latent behavioral phenotypes, animals were grouped using k-means clustering on the first three PCs. To determine the optimal number of clusters for k-means analysis, we computed silhouette scores across a range of *k* values. The silhouette method identified *k = 7* as the optimal solution, reflecting maximal separation between clusters with minimal within-cluster variance (Figure S1). While neighboring *k* values yielded similar overall structure, the *k = 7* solution most clearly dissociated high-hoarding, low-exploration, and exploratory-consumptive phenotypes, and was therefore used for all subsequent analyses. Each animal was assigned to a cluster based on Euclidean distance in PC space. Clusters were then interpreted as candidate behavioral phenotypes.

###### Cluster characterization

For each cluster, behavioral profiles were summarized by calculating the mean and standard deviation of all original behavioral variables. This allowed us to identify characteristic patterns of hoarding, exploratory behavior, and food consumption associated with each phenotype. Additionally, cluster membership was cross-tabulated with strain and sex to assess whether phenotypes were strain- or sex-dependent. ***Visualization*.** 3D PCA plots were generated using the first three PCs, with points colored according to cluster membership, to visually assess the separation of behavioral phenotypes. Variable contribution plots (correlation circles) were also created to highlight which behavioral measures contributed most strongly to each PC. Heatmaps of squared loadings were also generated to provide a comprehensive, quantitative view of variable contributions across the first seven PCs.

##### DeepLabCut velocity and posture

To visualize locomotor patterns, the horizontal (X-axis) position of the nose was tracked for each frame of the recording using Python. Data processing and plotting were performed with pandas, NumPy, Matplotlib, and Seaborn. For each animal, the nose X-position was cleaned by interpolating missing points and excluding frames with a likelihood below 0.9. The X-axis corresponds to the length of the arm in the testing apparatus, so the nose X-position reflects the animal’s progress along the arm over time. Steeper slopes in the X-position plots indicate faster movement, while flatter segments indicate slower movement or pauses. To summarize group-level locomotor dynamics, mean nose X-position per frame was calculated for each strain, sex, and arm. Eight subplots were generated (one per strain × sex × arm) showing the mean trajectory over time with shaded areas representing ±1 standard deviation. This approach provided a clear visual representation of speed, movement patterns, and time required to traverse the arm for each experimental group.

All statistical analyses of pose estimations were conducted in R. LMMs were used to examine the effects of strain (Wistar, Kyoto) and sex (male, female) on behavioral measures, with animal identity included as a random intercept to account for repeated observations within subjects. Models were fitted using the *lme4* package,^82^ and significance testing of fixed effects was carried out using the *lmerTest* package,^83^ which applies Satterthwaite’s approximation for degrees of freedom.

For locomotor activity, mean velocity was modeled as the dependent variable:

meanvelocity∼strain×sex+(1|ratID)

For posture, curvature angle was modeled as the dependent variable:

backcurvatureangle∼strain×sex+(1|ratID)

Back posture was measured to assess whether Kyoto rats exhibited more stretch-attend and stretch-approach posture, a body posture associated with heightened anxiety,^35^ compared to Wistar rats. This allowed us to quantify subtle anxiety-related differences in how animals navigated the apparatus.

Pairwise comparisons between sexes within each strain were performed using estimated marginal means with the *emmeans* package.^84^ Model assumptions were assessed by examining residuals; Shapiro–Wilk tests indicated no significant deviation from normality for either velocity (W = 0.987, p = 0.087) or curvature angle (W = 0.996, p = 0.871), supporting the use of LMMs.
