# Supplementary material for: Divergent behavioral strategies in Wistar and Wistar-Kyoto rats in a naturalistic task reveal mood disorder phenotypes
Source: iScience. 2026 Mar 21;29(4):115459. doi: 10.1016/j.isci.2026.115459 (PMC13084746; doi:10.1016/j.isci.2026.115459)
Supplement: Document S1. Figure S1 and Tables S1 and S2 [file mmc1.pdf]

iScience, Volume *29*

Supplemental information

Divergent behavioral strategies in Wistar and Wistar-Kyoto rats in a naturalistic task reveal mood disorder phenotypes

Jackson R. Ham and Robert J. McDonald

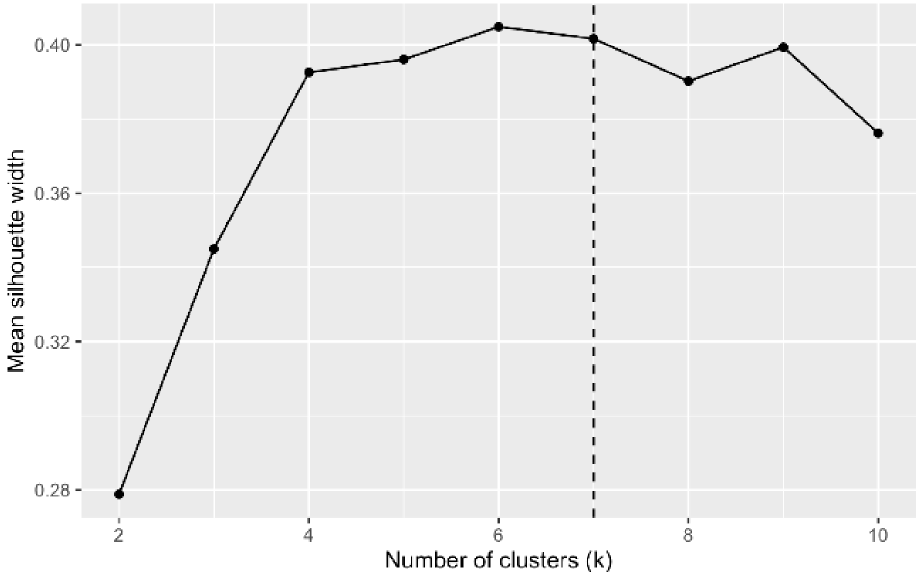

Supplementary Figure 1. Silhouette analysis for k-means clustering.
Average silhouette width across candidate cluster numbers shows a plateau of optimal solutions between k = 5 and 7. The k = 7 solution was selected to maximize separation while preserving distinct behavioral phenotypes.

Supplementary Table 1. Summary of statistical analyses. Summary of statistical tests for all panels in Figures 1, 2, and 4. Two-way ANOVAs examined effects of strain, sex, and strain × sex interactions. Pearson correlations were conducted within each strain–sex group. Linear mixed models (LMMs) were used for kinematic measures. Test statistics, model terms, and *P* values are reported.

Statistical analyses					
**Figure 1**					
**Panel**	**Comparison**	**Test**	**Effects**	**Model**	**P value**
B	Number of almonds eaten	2-way ANOVA	Strain × sex	F (1, 75) = 4.290	P=0.0418
			Sex	F (1, 75) = 0.1911	P=0.6633
			Strain	F (1, 75) = 27.74	P<0.0001
C	Time eating in the open	2-way ANOVA	Strain × sex	F (1, 75) = 0.6106	P=0.4370
			Sex	F (1, 75) = 0.008828	P=0.9254
			Strain	F (1, 75) = 2.007	P=0.1607
D	Time carrying food	2-way ANOVA	Strain × sex	F (1, 75) = 4.008	P=0.0489
			Sex	F (1, 75) = 0.3090	P=0.5800
			Strain	F (1, 75) = 31.89	P<0.0001
E	Number of almonds hoarded	2-way ANOVA	Strain × sex	F (1, 75) = 7.803	P=0.0066
			Sex	F (1, 75) = 0.8825	P=0.3505
			Strain	F (1, 75) = 11.77	P=0.0010
F	Time eating in the middle	2-way ANOVA	Strain × sex	F (1, 75) = 7.770	P=0.0067
			Sex	F (1, 75) = 0.2192	P=0.6410
			Strain	F (1, 75) = 3.208	P=0.0773

Correlations					
**Panel**	**Comparison**	**Test**	**Strain × sex**	**Model**	**P value**
**n/a**	Number of almonds hoarded x time spent in open arms	Pearson	Kyoto female	*r*(19) = 0.610	0.0033
**n/a**	Number of almonds hoarded x time spent in open arms	Pearson	Wistar female	*r*(22) = 0.016	0.942

**n/a**	Number of almonds hoarded x time spent in open arms	Pearson	Kyoto male	*r*(14) = 0.511	0.0018
**n/a**	Number of almonds hoarded x time spent in open arms	Pearson	Wistar male	*r*(16) = 0.470	0.0491

Figure 2

**Panel**	**Comparison**	**Test**	**Effects**	**Model**	**P value**
A	Time spent in open arms	2-way ANOVA	Strain × sex	F (1, 75) = 1.604	P=0.2092
			Sex	F (1, 75) = 4.066	P=0.0473
			Strain	F (1, 75) = 2.011	P=0.1603
B	Time spent in middle box	2-way ANOVA	Strain × sex	F (1, 75) = 1.593	P=0.2109
			Sex	F (1, 75) = 4.037	P=0.0481
			Strain	F (1, 75) = 2.002	P=0.1613
C	Number of middle chamber exits	2-way ANOVA	Strain × sex	F (1, 75) = 1.300	P=0.2578
			Sex	F (1, 75) = 7.484	P=0.0078
			Strain	F (1, 75) = 57.46	P<0.0001

Figure 4

**Panel**	**Comparison**	**Test**	**Effects**	**Estimate**	**Std. error**	**t value**	**P value**
C	Mean velocity	LMM	Intercept	285.561	27.822	10.264	P<0.0001
			Strain × sex	-7.565	55.644	-0.136	P=0.89283
			Sex	-17.456	39.346	-0.444	P=0.66071
			Strain	118.682	39.346	3.016	P=0.00539
E	Back angle	LMM	Intercept	144.085	1.666	86.483	P<0.0001
			Strain × sex	5.925	3.307	1.791	P=0.0841
			Sex	-4.5	2.342	-1.922	P=0.0649
			Strain	-1.786	2.35	-0.76	P=0.4536

Supplementary Table 2. Cluster characteristics across behavioral and feeding measures.
Clusters were defined based on behavioral and eating-related variables. For each cluster, the number of animals per strain and sex (Kyoto female/male, Wistar female/male) is reported alongside summary statistics (mean ± SD) for activity (exits), spatial behavior (time in open, right, left, and middle zones), and eating-related measures (carrying, eating in middle and open areas, total consumption, hoarding, body weight, and proportion eaten). Values represent cluster-level averages.

Cluster	Kyoto F	Kyoto M	Wistar F	Wistar M	Exits	Open	Right	Left	Middle	Carry	Eat Middle	Eat Open	Total Eaten	Hoarded	Weight	Proportion Eat
1	12	5	0	0	26.00 ± 3.94	918.69 ± 176.62	37.71 ± 20.28	32.89 ± 12.00	35.02 ± 9.85	39.41 ± 5.70	199.01 ± 93.25	16.71 ± 29.54	1.32 ± 0.40	9.88 ± 0.49	237.37 ± 65.41	0.13 ± 0.04
2	1	2	0	0	6.33 ± 1.53	69.07 ± 51.36	9.23 ± 6.16	10.56 ± 6.32	243.70 ± 55.30	0 ± 0	0 ± 0	0 ± 0	0 ± 0	0 ± 0	235.00 ± 53.70	0 ± 0
3	6	6	0	0	21.50 ± 5.28	358.14 ± 117.08	18.99 ± 8.73	15.19 ± 4.65	69.46 ± 21.01	29.93 ± 8.75	264.79 ± 112.76	8.99 ± 31.14	1.31 ± 0.60	7.83 ± 2.69	251.03 ± 65.21	0.21 ± 0.19
4	1	0	3	4	37.25 ± 6.54	821.75 ± 168.43	21.98 ± 6.05	22.70 ± 5.18	28.35 ± 9.64	30.60 ± 7.45	408.11 ± 87.33	29.13 ± 31.11	3.41 ± 1.21	8.88 ± 1.36	375.29 ± 142.15	0.38 ± 0.12
5	1	3	10	5	29.00 ± 10.06	560.02 ± 104.72	22.19 ± 5.75	19.63 ± 7.55	47.73 ± 19.21	12.24 ± 7.99	181.21 ± 74.09	18.77 ± 31.79	1.57 ± 0.56	3.00 ± 1.63	326.94 ± 116.63	0.61 ± 0.26
6	0	0	3	6	33.67 ± 5.92	575.66 ± 94.42	17.67 ± 4.13	15.01 ± 3.43	38.98 ± 11.83	16.88 ± 5.49	349.55 ± 60.33	3.54 ± 5.49	3.78 ± 0.96	5.33 ± 1.50	424.28 ± 95.35	0.74 ± 0.18
7	0	0	7	1	50.00 ± 12.00	892.95 ± 170.66	18.82 ± 4.68	18.33 ± 5.54	19.36 ± 7.97	9.25 ± 7.28	115.22 ± 56.97	102.23 ± 70.10	1.56 ± 0.44	2.63 ± 2.26	287.88 ± 65.27	0.77 ± 0.24
